# Evaluation of Technical Condition and Durability of Wooden Shaft Guides with Application of Non-Destructive and Semi-Destructive Testing Methods

**DOI:** 10.3390/ma15144769

**Published:** 2022-07-07

**Authors:** Rafał Pasek, Justyna Jaskowska-Lemańska, Daniel Wałach, Tomasz Rokita, Paweł Kamiński

**Affiliations:** 1 Faculty of Mechanical Engineering and Robotics, AGH University of Science and Technology, Adama Mickiewicza Ave. 30, 30-059 Krakow, Poland; rpasek@agh.edu.pl (R.P.); rokitom@agh.edu.pl (T.R.); 2 Faculty of Civil Engineering and Resource Management, AGH University of Science and Technology, Adama Mickiewicza Ave. 30, 30-059 Kraków, Poland; walach@agh.edu.pl (D.W.); pkamin@agh.edu.pl (P.K.)

**Keywords:** NDT methods, SDT methods, wooden shaft guides, durability of shaft

## Abstract

This article addresses the issue of the durability of mining shaft equipment elements. Shafts as a transport route are one of the most exploited parts of a mine. Consequently, their components are exposed to high mechanical stresses, which cause the deterioration of their mechanical properties. In the case of shafts with timber components, elements such as the shaft guides are evaluated on a purely macroscopic basis and are often unnecessarily replaced. This paper presents the possibilities for the application of non-destructive methods (ultrasound and laser scanning) and semi-destructive methods (sclerometric and drill resistance tests). The experimental results suggest that it was possible to derive correlations between penetration depth and drill resistance tests with bulk density. However, these tests were not directly correlated with flexural strength. The ultrasound studies did not indicate a significant relationship with the physical or mechanical properties. In contrast, the method of comparing the variation (wear) in the tested guides using 3D laser scanning demonstrated a high accuracy; moreover, this method is independent of factors that may affect the results of penetration depth or drill resistance measurements. The application of non-destructive and semi-destructive tests for the determination of the physical and mechanical properties of timber elements of mine shafts’ equipment may enable the detection of a defect earlier or extend the service life of elements, hence limiting the downtime of shaft operation related to the replacement of elements.

## 1. Introduction

### 1.1. Wood Testing Methods

Mine shafts are of fundamental importance for the proper and safe functioning of underground mines. For this reason, the shaft casing and its equipment have to meet high requirements, which are periodically verified by appropriate testing and assessment of their technical condition in accordance with standards and regulations. These regulations in turn oblige mine inspectors to use increasingly advanced measuring methods, which precisely determine the type and level of damage to the shaft lining, reinforcement, and other equipment, which has a significant impact on the shaft stability assessment.

Timber, as a construction material, shows a high durability in favorable conditions. However, in typical conditions, it is exposed to numerous factors affecting its longevity. In addition, the specific operation of the shaft components exposes the material not only to varying climatic conditions but also to varying loads [1]. Thus, the frequent technical condition assessment of wooden constructions is necessary. Such evaluations are conducted during modernization as well as during ongoing inspections of all facilities, of both the standard building type and underground construction structures [2,3,4]. Such assessments are particularly crucial in historic sites, such as the Wieliczka salt mine, which is on the UNESCO World Heritage List. However, the evaluation of wooden elements of mine shaft lining and equipment usually comprises only macroscopic examination. As a result, numerous elements are replaced despite their good technical condition. In recent years, several documents have been created presenting preferable methods for the evaluation of timber conditions in constructions [5,6].

Structural element testing comprises destructive testing (DT), non-destructive testing (NDT), and semi-destructive testing methods (SDT). Destructive testing methods require the collection of a significant amount of samples, which might be impossible in the case of operating objects. Thus, the current standards recommend the use of NDT and SDT methods [5,7,8].

Non-destructive testing methods of constructions in underground engineering most often include visual assessment and surveying techniques [9,10]. More rarely used are techniques based on electromagnetic wave propagation [11] as well as X- and gamma-rays methods [12,13], which are successfully applied in the case of testing typical objects, while in the case of underground structures they rather refer to other materials such as steel or concrete [14,15]. In contrast, semi-destructive methods are characterized by a small influence on an examined object—only small defects occur as a result of the test. In terms of mine shaft constructions, in which a high level of safety has to be maintained, these methods appear to be implemented increasingly, often due to their higher accuracy in comparison to NDT tests. This group of methods comprises penetrance tests (hardness tests, drill resistance measurement) [15,16], pull-off testing methods [17], small-samples collecting methods [16,18]. The assessment of the technical condition of timber constructions usually comprises the use of both non-destructive and semi-destructive methods [19,20]—which are partially used in the mining industry, but in relation to other materials [15]. In recent years, the rapid development of non-destructive and semi-destructive testing methods has been observed, as well as their application in various industries. However, it should be noted that the test results might be influenced by numerous factors, such as wood species, defects, humidity, temperature, age, or microbial agents [21,22]. The influence of different factors on test methods and some of wood species are presented in [23,24,25,26,27].

This work presents the application of modern methods of physical and mechanical parameter (bulk density and flexural strength) evaluation for wooden elements of mine shafts.

### 1.2. Characteristics of Wieliczka Salt Mine Shafts

The Wieliczka Salt Mine is one of the oldest companies in the whole of Europe and one of the biggest tourist attractions in Poland, with over a million visitors a year (data before the outbreak of the COVID-19 pandemic). To simultaneously handle thousands of tourists and mine operation (comprising the reconstruction and maintenance of historic workings and the liquidation of the others), three hoisting systems in three different shafts are used. Two of them are equipped with the wooden guidance of conveyances [28].

The Kinga shaft, the main mine operations shaft, is equipped with a stiff, side guidance of conveyances, which are two double-compartment cages. The guides are made of solid pinewood or laminated larch wood. The dimensions of their cross-section are 140 × 130 mm and the length of a single beam is 6 m. The guides are attached to wooden or steel buntons at an interval of 3 m. The material of the guides and buntons is dependent on their installation time, as the shaft was sunk in the 19th century and its last modernization was conducted in the 1970s. Since that time, numerous minor renovations have been conducted.

The Daniłowicz shaft, sunk in the 17th century, now serves the tourist traffic. It is equipped with a hoisting system comprising a cage and a counterweight. Both the cage and the counterweight are guided with the use of stiff wooden guides. The dimensions of the guides are 160 × 140 mm. The loads acting on the guides and caused by the conveyances obtained in the mine measurements are presented in Table 1. These forces affect the structure and surface of the guide material, causing deformation and degradation.

## 2. Materials and Methods

### Non-Destructive and Semi-Destructive Testing Methods

To assess the applicability of non-destructive and semi-destructive testing methods for periodic evaluation of the condition state of wooden guides in mine shafts, laboratory tests were conducted. The tests comprised six elements of pinewood guides used in the shafts of Wieliczka salt mine (Kinga and Daniłowicz) and five reference elements. The lengths of the test samples were between 1000 and 1500 mm. Reference samples were selected on the basis of density and visual assessment (similar amount of jars and knots). During the test, all samples were in an air-dried state. The parameters of the samples are presented in Table 2.

**Figure 1:**
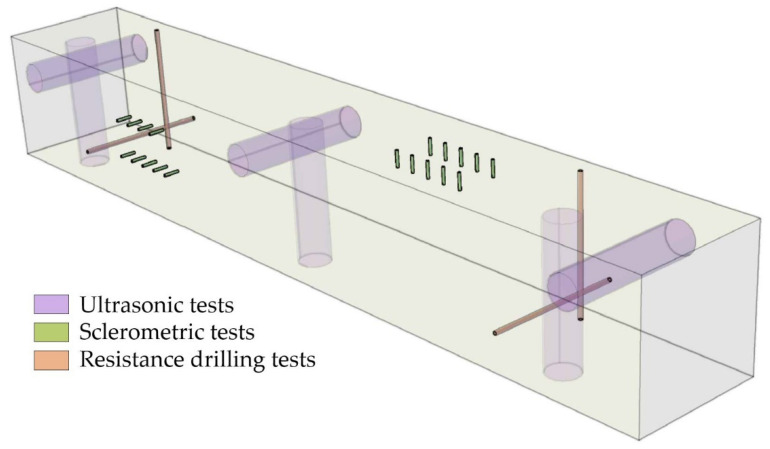
A diagram of a sample.

The test procedure comprised a macroscopic assessment (location of defects to eliminate their influence on results), density evaluation, ultrasonic, sclerometer, and drill resistance tests. The direction of measurements was perpendicular to the wood fibers. Typical destructive flexural strength tests were also conducted. Moreover, X-ray computed tomography was used to map the structure destruction of the guide surface. Figure 1 presents a diagram of a sample with the tested area marked.

Ultrasonic tests were conducted with the use of the Pundit Lab set by Proceq with 54 kHz frequency transducers. Before every test, the device was calibrated with a calibration rod with a passing time of the ultrasonic wave equal to 25.4 μs. A chemically neutral polyacrylic gel was used as a coupling agent. Both transducers were covered with gel before each test. Four defect-free measurement points were selected for each sample, two on each side of the sample. Three measurements were conducted for each measurement point of each sample. The results of the test comprise the average values of the speed of ultrasonic wave (v) passing through the sample.

A Woodtester by Novatest with an impact energy of 2.4 J was used in sclerometer testing. It was equipped with 60 HRC steel needles with a diameter of 2.5 mm with a conical tip and length of 50 mm, and a dial gauge with an accuracy of up to 0.01 mm. Measurements were conducted in the horizontal position. One result of the test is the penetration depth of the needle after one (PD_1_) and two (PD_2_) impacts. Three measurement fields were selected on each sample. Each of the fields was assessed as free from defects and was located on a different side of the sample. In each of them, nine measuring points were selected.

Drill resistance tests were conducted using the Resistograph 4453-s by RinnTech with a drill feed of 40 cm/min and a resolution of 1/100 mm. Such measurement allows the determination of average wood density. The torque required to maintain a constant drilling speed, corresponding to cutting resistance, was recorded during the test in relation to the drilling depth. The test result is a dimensionless value RM, which is a quotient of the area below the cutting resistance diagram and depth of the hole [29]. Four tests were conducted for each sample (two on each side) in areas selected as defect-free.

Flexural strength tests were conducted with a Walter+Bai hydraulic testing machine. A three-point flexural test was used. Tests were performed on full-size elements extracted from the shaft with dimensions according to Table 2. The area of the sample cross-section was measured directly before the test with an accuracy of up to 0.1 mm. The displacement velocity was equal to 0.01 mm/s.

Static flexural strength was calculated with an accuracy of up to 0.1 MPa according to the following formula:

f_m_ = (l·F_max_)/(4·W) (1)

where:

f_m_—static flexural strength in three-point flexural test, MPa;
l—distance between the supports, l = 900 mm;
F_max_—maximum load (destructive force), N;
W—section modulus, mm^3^.

X-ray computed tomography was used to map the geometric deviations of the guides after a given time of their service in the shaft. A GE v|tome|x M scanner with a resolution of 150 µm was used. The parameters of the lamps were set at the level of 100 kV and 100 µA. A total of 3000 images were taken in a single test. Data reconstruction was conducted with specialized software. Reference samples were not subject to testing. The obtained geometries of the samples were compared with their nominal shapes, generated as 3D CAD models. The nominal shapes have been defined as cuboids with cross-sectional dimensions adjusted to the steel elements of their mounting in the shaft, i.e., to their original dimensions. It should be emphasized that this is an idealized model, but due to the technical requirements in the shafts, the acceptable dimensional deviations for newly-installed elements are very small. The nominal dimensions of the guides are presented in Table 3.

**Figure 2:**
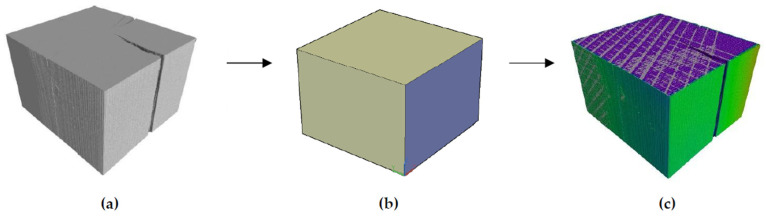
Procedure of geometric deviation mapping of guide samples; (**a**) X-ray image, (**b**) CAD model, (**c**) deviation map on the 3D model.

A schematic diagram of geometric deviation mapping is presented in Figure 2.

It should be noted that there are other methods of determining geometric models for the purpose of X-ray computed tomography, such as a point cloud obtained in 3D laser scanning or using photogrammetry [30].

## 3. Results

### Traditional DT, NDT, and SDT Testing Methods

The average values of parameters obtained in destructive, non-destructive, and semi-destructive tests are presented in Table 4. The findings of the destructive tests apply to the whole sample, while the results of the non-destructive and semi-destructive tests refer to two different cross-sections.

The examined guide samples were characterized by a flexural strength between 30 and 50 MPa, which is a relatively low value considering the small number of knots and good technical condition of the guides according to macroscopic assessment. However, the strength condition for the guides was still fulfilled.

The highest velocity of passing was recorded for sample two (which was made of laminated timber) in the direction perpendicular to the lamination. The value of this velocity was equal to 2456.2 m/s. In most of the remaining cases, the velocity did not exceed 2000.0 m/s. The lowest velocity was equal to 1691.4 m/s in the case of sample five.

**Figure 3:**
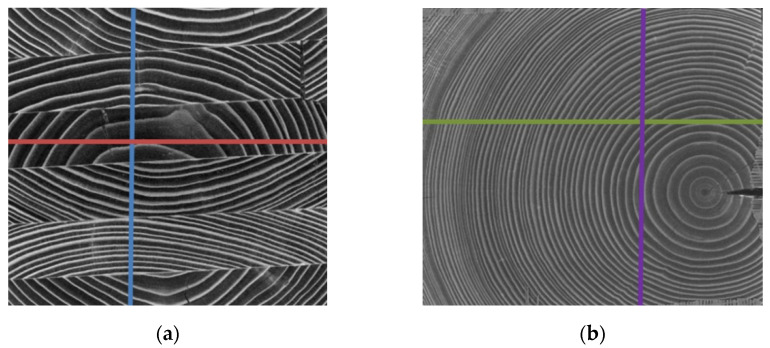

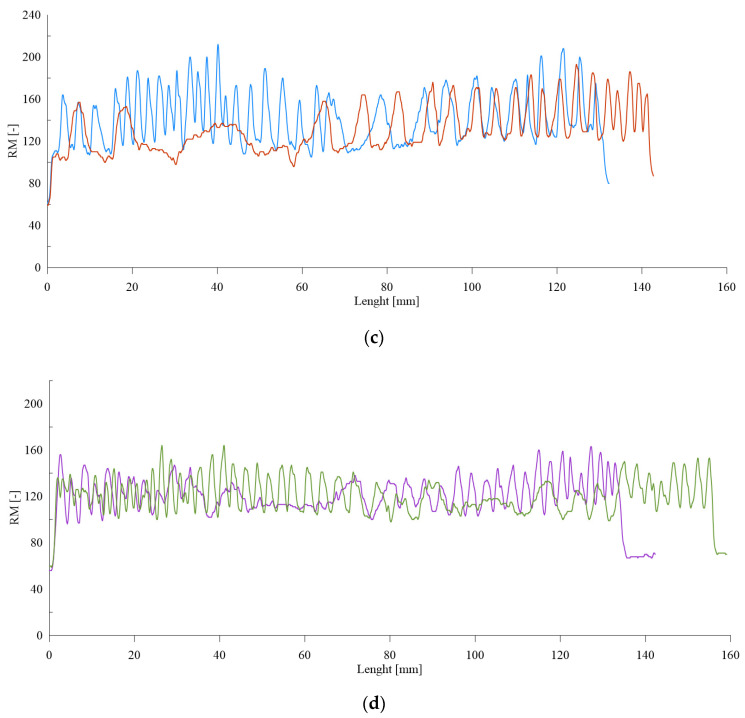
Results of drill resistance tests of samples two and six; (**a**) location of drilling lines in sample two, (**b**) location of drilling lines in sample six, (**c**) drill resistance diagram of sample two, (**d**) drill resistance diagram of sample six.

The smallest penetration depth was recorded for sample three, with the greatest for sample two in the direction perpendicular to the lamination. The average values of drill resistance were similar for all samples (except sample two). Similarly to other quantities, sample two was characterized as having the greatest drill resistance. Samples five and six were characterized as having the lowest drill resistance. Figure 3 presents a diagram of the drill resistance obtained for samples two and six.

**Figure 4:**
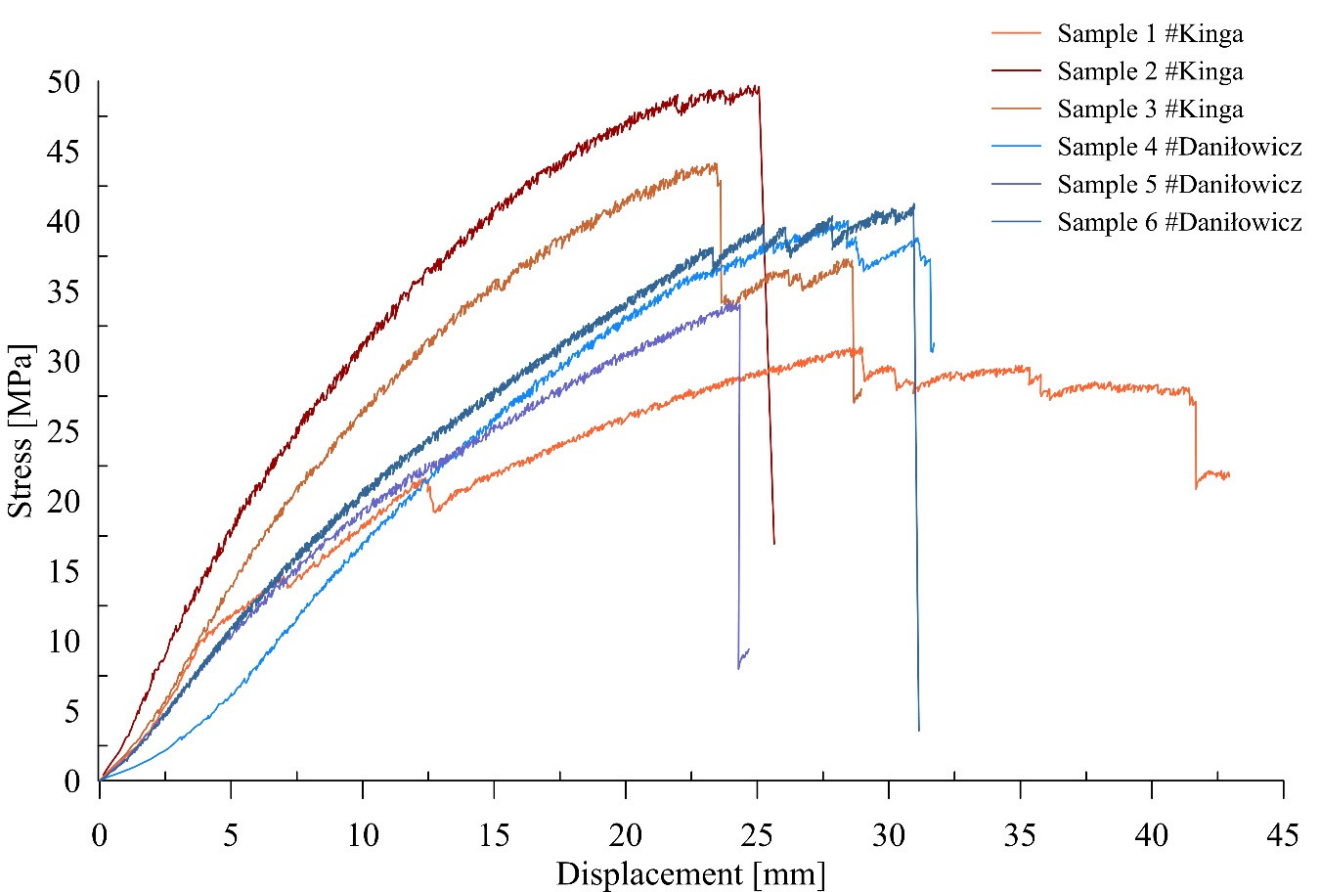
Stress-strain characteristics of samples in flexural strength tests.

Figure 4 presents the stress-strain characteristics of the samples in flexural strength tests. It can be observed that the laminated wood guide is characterized by the greatest strength and stiffness. The values of the deformation parameters of the Daniłowicz shaft guides are similar; in the case of the Kinga shaft guides, they differ from each other.

Table 5 presents the results of the reference samples’ tests. As the elements were not previously used in construction, average values of the measured quantities of non-destructive and semi-destructive tests were adopted as reference values.

The flexural strength of the reference samples was between 48 and 88 MPa, which is a greater value than in the case of samples of guides. The smallest penetration depth was recorded for sample three and the greatest for sample four. Similarly, sample four had the lowest ultrasonic wave velocity, equal to 1716 m/s. Sample five was characterized as having the greatest value of velocity of passing, equal to 2211 m/s. The value of drill resistance was between 124 and 152, the greatest value of which was measured for sample four.

**Figure 5:**
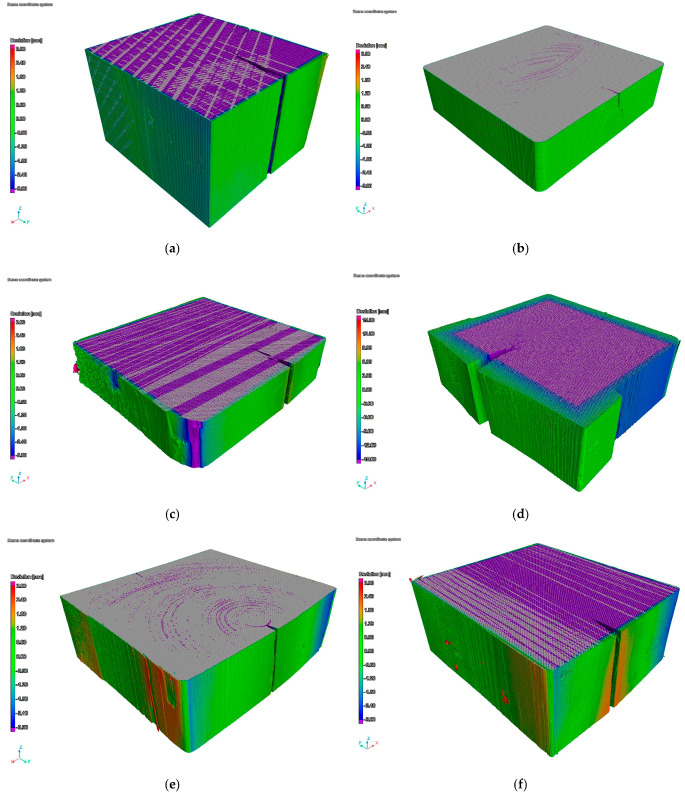
Images of changes in the shape of samples according to CT; (**a**) sample one; (**b**); sample two; (**c**) sample three; (**d**) sample four (larger scale); (**e**) sample five; (**f**) sample six.

The purpose of the CT analysis was a volumetric analysis and a comparison of the obtained shapes and dimensions of the samples and the CAD models. The results of the analysis are images of samples showing the differences between the dimensions of the models and samples. Color is a measure of the shape and dimensions deviation of the samples. The results of the test are shown in Figure 5.

**Figure 6:**
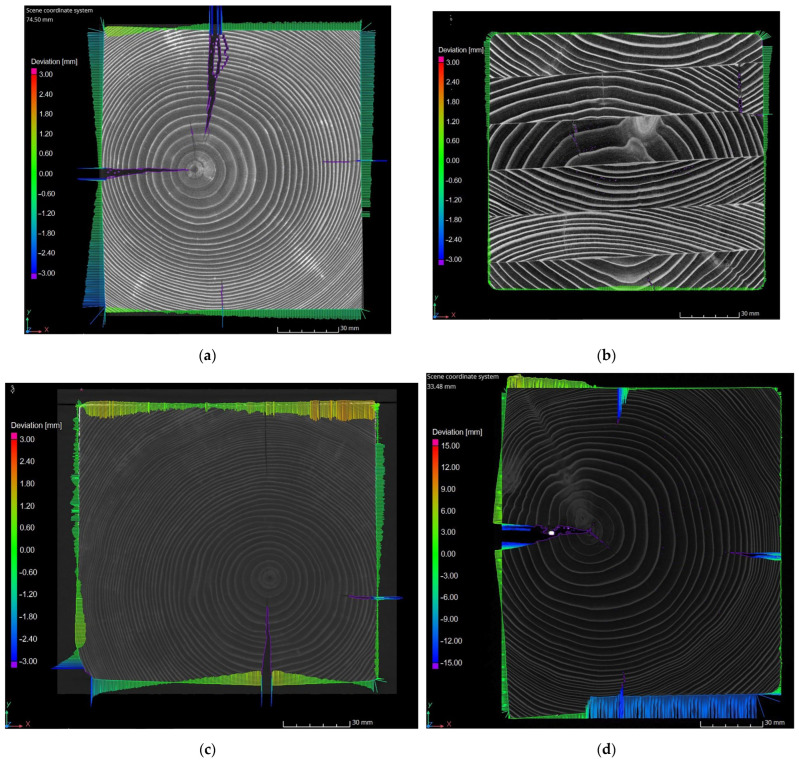

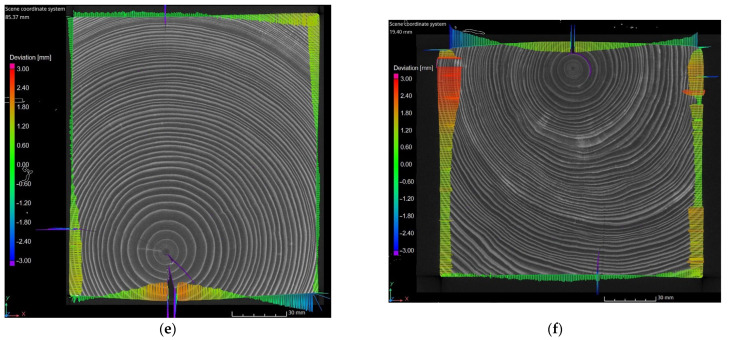
Bar charts of the deviations of the selected samples’ cross-sections; (**a**) sample one; (**b**) sample two; (**c**) sample three; (**d**) sample four (larger scale); (**e**) sample five; (**f**) sample six.

An analysis comprising bar charts showing shape variations was conducted for selected samples’ cross-sections. Positive values (bars inside a cross-section) are sections of the sample where the area of the measured surface is greater than surface of the CAD model, while negative values (bars outside a cross-section) are sections where the real area or the sample cross-section is smaller than the area of the CAD model. Sample four had the greatest values of the deviation, reaching 15 mm. The smallest deviation was measured for sample two. Other samples were characterized by a variable geometry of cross-sections; however, the values of their deviation did not exceed 3.0 mm. Bar charts of the deviations of the selected samples’ cross-sections are presented in Figure 6.

## 4. Discussion

In order to prove the applicability of non-destructive and semi-destructive testing methods for evaluation of the technical state of wooden guides, a number of tests and measurements were conducted, including tests of samples of guides used previously in mine shafts and reference samples (of similar parameters, including density, humidity, and amount of knots). In comparing the NDT and SDT results, the data obtained for sample two (laminated guide) were omitted, as the results of such tests in the case of laminated timber elements are strongly dependent on the properties of the slats.

**Figure 7:**
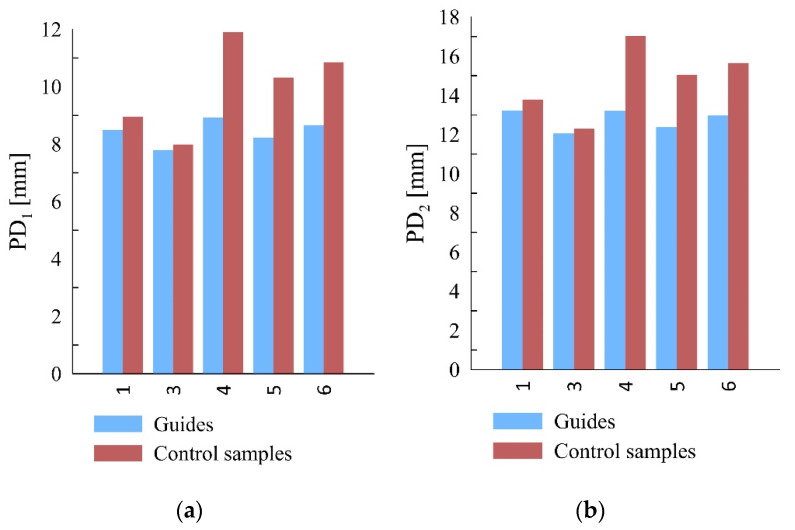
Comparison of sclerometric tests results of guide and reference samples; (**a**) single impact, (**b**) double impact.

Sclerometer tests, with both the single and double impact of the needle, proved a strong correlation between the penetration depth and density of the sample—the greater the density, the smaller the penetration depth. The analysis of this relationship for the guides provided a Pearson’s correlation coefficient of r = 0.93 for single impact and 0.95 for double impact. A comparable correlation strength was obtained for the control samples. However, it should be remembered that the tests were conducted on a limited number of samples. A strong correlation between penetration depth and volume density is confirmed by the literature [17,19]. However, the values of the penetration depths in the case of the guide samples were significantly smaller than in the case of the reference samples, by 1.6 mm for single impact and 2.0 mm for double impact on average. The variation in the results of samples four and six was greater than in the case of samples one and three. Smaller penetration depths might have a number of causes, such as the previous long-term operation of guides resulting in the hardening of the surface timber layer due to its compression caused by loads acting on the guide. It should be noted that the surface hardening of the guide has no relationship with flexural strength, and the relationship of penetration depth to flexural strength for the guides is negligible. Figure 7 presents a comparison of the sclerometric test results.

A comparison of the ultrasonic wave passing test results did not show any correlations between the values obtained for guide and reference samples (r < 0.3). The ultrasonic wave passing velocity of samples one and four was greater than its measured values for reference samples one and four (characterized by a similar density). In contrast, guide samples three, five, and six has lower values of passing speed than the corresponding reference samples. The results of the measurements do not comply with the correlation presented in literature data, which is an increase in the wave passing velocity with increasing density. However, it should be emphasized that this correlation is usually weak and a large number of samples is required to determine it.

**Figure 8:**
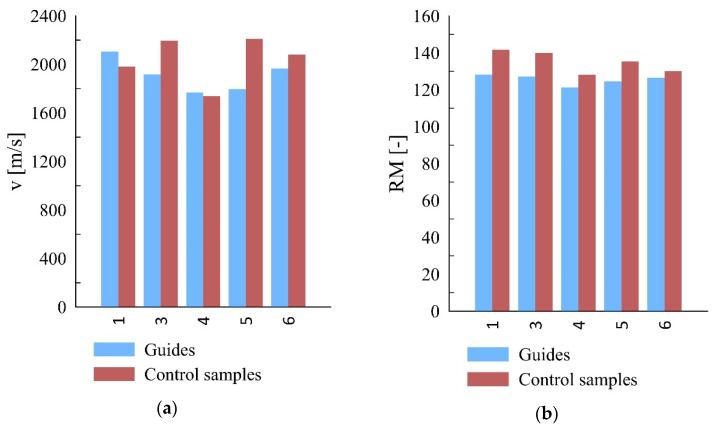
Comparison of ultrasonic and drill resistance test of guide and reference samples; (**a**) ultrasonic tests, (**b**) drill resistance tests.

The results of the drill resistance test revealed a relationship between density and drill resistance—the greater the density, the greater the drill resistance. In this case, the Pearson’s correlation coefficient was r = 0.62. An analysis of the average drill resistance values shows that in the case of reference samples, they are about 8% greater than for guide samples. It may be considered a confirmation of the thesis of the guides’ hardening (according to sclerometer tests) solely on the surface. The inside of the guide is subject to natural aging processes. Figure 8 presents a comparison of the average values of ultrasonic wave passing velocity and RM (drill resistance).

**Figure 9:**
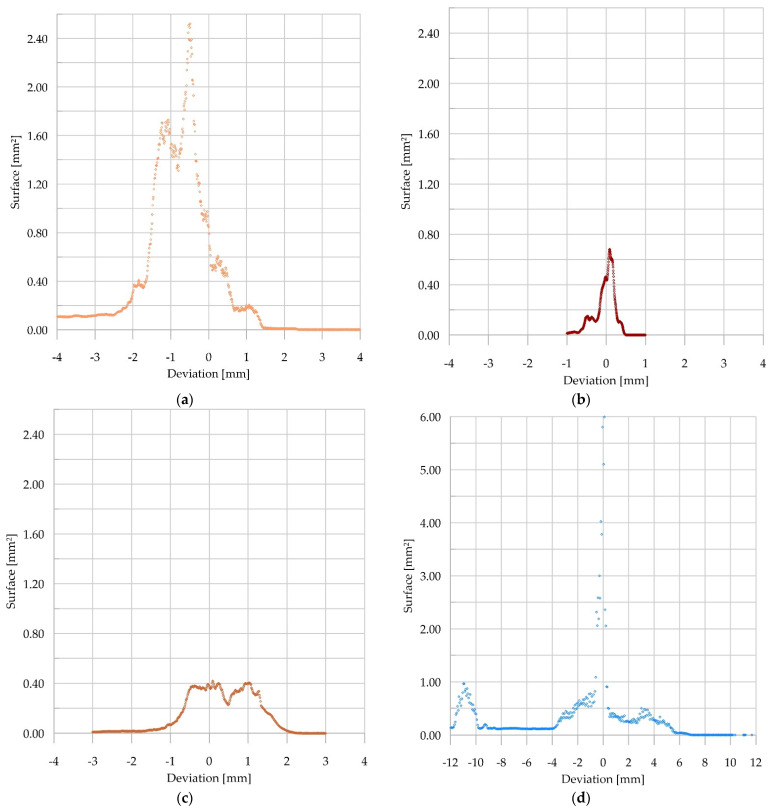

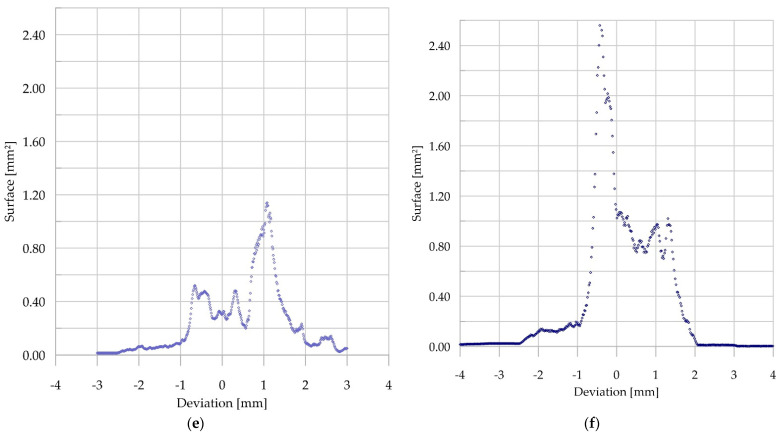
Distribution of deviation of guide samples; (**a**) sample one; (**b**) sample two; (**c**) sample three; (**d**) sample four; (**e**) sample five; (**f**) sample six.

In CT analysis, the quantity of geometric deviation of the samples was determined as a measure of the guides’ quality. Figure 9 presents the average area of the guide samples in a given distance from reference CAD models. The calculated values of the deviation refer to a section of the sample of 1.0 mm in height. It was assumed that the selected section of the sample is statistically repetitive. On both sides of the zero value, irregular falls of the diagram can be observed. This can be interpreted as an effect of defects, fractures (negative values), or distortion (positive values).

The negative value of the surface deviation of sample one (Figure 9a) was a result of the conveyance’s guide rollers’ influence. Most of the recorded deviations were in the range of −0.2 to −1.5 mm. The value of the deviation on the edge of the sample reached −2.8 mm. The peaks of the deviation values were caused by longitudinal fractures.

Sample two was characterized as having both the smallest number (Figure 9b) and value (Figure 6b) of the deviation. The obtained values of approximately 0.5 mm do not exceed the error margin. Such results prove the high durability of the laminated guide.

The values of deviation measured for sample three did not exceed 1.0 mm. However, it should be noted that both positive and negative values were obtained. This reflects the surface deformation shown in Figure 5c, which is an effect of conveyance loading at the shaft outset. Similarly to sample one, the maximum values of deviation were caused by longitudinal fractures and additionally by a rounded corner of the guide, which can be seen in Figure 5c.

Sample four, due to long-lasting loads caused by the slipping plate acting on the guide, was characterized as having the greatest values of deviation (Figure 5d). Its long-term operation resulted in a deviation reaching −11.0 mm, which can be seen in Figure 9d. Positive values of deviation were also recorded in the area of the longitudinal fracture of the sample, which indicates distortion.

Similarly to sample three, the values of deviation recorded for samples five and six were about 2.0 mm (in the case of sample six they locally reached 3.0 mm). It should be emphasised that both positive and negative values were recorded, which illustrates surface deviation as shown in Figure 5e,f. The maximum values of deviation were measured in areas of longitudinal fractures, similarly to samples one and three, and near the rounded corners of the guides.

## 5. Conclusions

An analysis of research NDT and SDT results shows that the correlation dependence formula may be derived for a certain object (e.g., a mine shaft or a set of shafts of the same function). However, it is not possible to apply the currently known correlation functions due to the significant differences that are obtained for elements subjected to shaft exploitation compared to new/reference elements. These differences are presumably a result of the element exploitation type and the associated surface hardness increase. Wood density may be a criterion of wood quality, and correlations between density and mechanical properties are widely recognized. A strong relationship between sclerometer test results and density was shown. Similarly, the results of drill resistance tests are in strong correlation with density. Such relationships are weaker in the case of flexural strength. However, in both cases it should be noted that the test sample was relatively small and in order to determine dedicated linear functions, the test sample would need to be increased. In the case of ultrasound wave velocity studies across fibers, no density or flexural strength relationships were indicated. In future research, ultrasound investigations along wood fibers could be considered.

The proposed method of comparison of deviations (wear) in the examined guides demonstrates a high accuracy and thus might be used in the real-life conditions of a mine. In such cases, it is necessary to gather data on the current guides’ geometry using, e.g., 3D laser scanning or photogrammetry. These methods are broadly used for data acquisition in civil engineering and offer the possibility of the completely portable imaging of the studied elements. The resulting data allow for an accurate analysis of the deviation using the research method presented in this paper.

Nowadays, the evaluation of timber guides in mine shafts consists exclusively of visual assessment. This often results in the replacement of technically sound elements that contain only superficial defects that do not affect the structural stability. The modernization of the assessment methods of timber elements in mine shafts should be conducted, since the knowledge of the current physical and mechanical parameters of timber used in mine shafts may prolong the service life of a given element, which is extremely beneficial from the point of view of mine operation (no interruptions in the main communication passage). In extreme cases, such an assessment may also allow for earlier detection of the hazards associated with component failure. Such damage may occur due to the specific microclimatic conditions in the shaft. As a general rule, a low-humidity or completely waterlogged environment will be much better for the timber than an environment with frequent variations in humidity. In mine shafts, depending on their function, the microclimatic conditions may be different [31], which will influence the durability of the applied timber as well as the results of non-destructive or semi-destructive tests [21].

## Figures and Tables

**Table 1:** Loads acting on the guides installed in Daniłowicz shaft.

Guide		Front Load [kN]	Side Load [kN]
cage	west	5.0	3.3
	east	6.0	3.2
counterweight	west	5.7	6.4
	east	7.1	7.1

**Table 2:** Parameters of the test samples.

No.	Sample Origin	Sample Type and Age	Average Sample Cross-Section Dimensions [mm]	Average Sample Density in Air-Dry State [kg/m^3^]
1	#Kinga	solid, ~10 years	128 × 139	489.6
Ref 1	reference		125 × 125	491.1
2	#Kinga	laminated, ~5 years	130 × 140	556.4
3	#Kinga	solid, 15–20 years	124 × 135	631.4
Ref 3	reference		125 × 125	635.2
4	#Daniłowicz	solid (counterweight), ~10 years	132 × 147	454.9
Ref 4	reference		125 × 125	458.3
5	#Daniłowicz	solid (counterweight), ~2 years	138 × 155	583.4
Ref 5	reference		125 × 125	589.2
6	#Daniłowicz	solid (cage), ~5 years	138 × 155	495.3
Ref 6	reference		125 × 125	494.6

**Table 3:** Nominal dimensions of samples.

No.	Nominal Dimension No. 1 [mm]	Nominal Dimension No. 2 [mm]	Age [Years]
1	130	130	10
2	130	140	5
3	125	125	15–20
4	160	160	10
5	135	135	2
6	155	155	5

**Table 4:** Average results of destructive, non-destructive, and semi-destructive tests.

No.	Cross-Section	Density [kg/m^3^]	PD_1_ [mm]	PD_2_ [mm]	v [m/s]	RM [-]	f_m_ [MPa]
1	A-A	489.6	8.43	13.29	2116.7	129.99	31.01
	B-B		8.55	13.17	2095.8	126.56	
2	A-A	556.4	9.99	14.97	2456.2	140.98	49.91
	B-B		8.86	15.06	1827.3	131.28	
3	A-A	631.4	7.54	11.71	1971.4	128.22	44.34
	B-B		8.05	12.41	1863.3	126.21	
4	A-A	454.9	8.96	13.28	1755.6	120.75	40.19
	B-B		8.90	13.13	1780.3	122.37	
5	A-A	583.4	8.58	12.57	1691.4	121.98	34.35
	B-B		7.87	12.21	1899.8	127.28	
6	A-A	495.3	8.18	12.74	1907.6	130.08	41.25
	B-B		9.15	13.22	2021.9	122.94	

PD—penetration depth, v—velocity of the ultrasonic wave passage, RM—drill resistance, f_m_—flexural strength.

**Table 5:** Average results of destructive, non-destructive, and semi-destructive tests of reference samples.

No.	Density [kg/m^3^]	PD_1_ [mm]	PD_2_ [mm]	v [m/s]	RM [-]	f_m_ [MPa]
Ref 1	491.1	8.95	13.7850	1982.14	141.74	68.05
Ref 2	635.2	7.99	12.3057	2195.99	139.99	88.12
Ref 3	458.3	11.90	17.0322	1738.89	128.17	51.22
Ref 4	589.2	10.32	15.0548	2211.37	135.351	74.14
Ref 5	494.6	10.85	15.6519	2080.40	130.098	48.76
Ref 6	491.1	8.95	13.7850	1982.14	141.74	68.05

PD—penetration depth, v—velocity of the ultrasonic wave passage, RM—drill resistance, f_m_—flexural strength.

## Data Availability

The data that support the findings of this study are available from the corresponding author upon request.
